# Supplementary material for: A neural network model of hippocampal contributions to category learning
Source: eLife. 2023 Dec 11;12:e77185. doi: 10.7554/eLife.77185 (PMC10712951; doi:10.7554/eLife.77185)
Supplement: Figure 3—source code 1. [file elife-77185-fig3-code1.zip › 18-01-2022-RA-eLife-77185/Figure3Code.html]

A neural network model of hippocampal contributions to category learning


# A neural network model of hippocampal contributions to category learning

## Figure 3 - Source Code

This is an R code used to create Figure 3 for the paper “A neural
network model of hippocampal contributions to category learning”.

https://www.biorxiv.org/content/10.1101/2022.01.12.476051v1

```
# Load libraries
library(readxl)
library(dplyr)
library(ggplot2)
library(reshape2)
library(RColorBrewer)
library(ggpubr)
library(grid)
```

```
# Set working directory
#setwd("C:/Users/hip-cat/results")

# Load data
data_fig3 <- as.data.frame(read.csv("Figure 3 - Source Data 1.csv"))

# Subset data for human performance on unique feature recognition  
h_ufr <- data_fig3 %>%
  filter(condition == "Humans" & test_type == "unique")

# Subset data for human categorization performance  
h_cat <- data_fig3 %>%
  filter(condition == "Humans" & test_type == "cat")

# Subset model accuracy data for unique feature recognition 
m_ufm <- data_fig3 %>%
  filter(condition != "Humans") %>%
  filter(test_type == "unique")

# Subset model data for categorization performance 
m_cat <- data_fig3 %>%
  filter(condition != "Humans") %>%
  filter(test_type == "cat")

# Subset model data for generalization performance 
m_gen <- data_fig3 %>%
  filter(condition != "Humans") %>%
  filter(test_type == "gen")
```

```
# plot theme specs 
plot_specs <- 
    theme_bw() +
    theme(panel.grid.major = element_blank(), 
        panel.grid.minor   = element_blank(),
        panel.border       = element_blank(),
        axis.line          = element_line(colour = "black"),
        axis.text          = element_text(size = 15),
        axis.title         = element_text(size = 20),
        plot.title         = element_text(size = 25), 
        legend.text        = element_text(size = 15),
        legend.title       = element_blank())
```

## Figure 3a. Human unique feature recognition

Human performance on unique features across training in Schapiro,
McDevitt et al. (2017).

```
ggplot(h_ufr, aes(x = trial, y = accuracy, color = test_type)) +
  geom_point() +
  geom_errorbar(aes(y    = accuracy, 
                    ymin = accuracy - standard_error, 
                    ymax = accuracy + standard_error, 
                    width = 0.1)) +
  geom_smooth(method = lm, 
              se = FALSE) +
  geom_hline(yintercept = 0.25, 
             color      = "grey", 
             linetype   = "dashed", 
             size       = 2) +
  scale_x_continuous(breaks = c(16, 32, 48, 64, 80, 96, 112, 128)) +
  scale_y_continuous(breaks = c(0, 0.25,  0.5,  0.75, 1),
                     limits = c(0.2, 1)) +
  labs(x     = "Trial", 
       y     = "Accuracy", 
       title = "Human unique feature performance") +
  scale_color_manual(values = c('#1b9e77')) + 
  plot_specs +
  theme(legend.position = "none")
```

```
## Warning: Using `size` aesthetic for lines was deprecated in ggplot2 3.4.0.
## ℹ Please use `linewidth` instead.
## This warning is displayed once every 8 hours.
## Call `lifecycle::last_lifecycle_warnings()` to see where this warning was
## generated.
```

```
## `geom_smooth()` using formula = 'y ~ x'
```

## Figure 3b. Human categorization performance

Human performance on categorization across training in Schapiro,
McDevitt et al. (2017).

```
ggplot(h_cat, aes(x = trial, y = accuracy, color = test_type)) +
  geom_point() +
  geom_errorbar(aes(y    = accuracy, 
                    ymin = accuracy - standard_error, 
                    ymax = accuracy + standard_error, 
                    width = 0.1)) +
  geom_smooth(method = lm, 
              formula = y ~ poly(x, 2), 
              se = FALSE) +
  geom_hline(yintercept = 0.33, 
             color      ="grey", 
             linetype   = "dashed", 
             size       = 2) +
  scale_x_continuous(breaks = c(16, 32, 48, 64, 80, 96, 112, 128)) +
  scale_y_continuous(breaks = c(0, 0.25,  0.5,  0.75, 1),
                     limits = c(0.2, 1)) +
  labs(x     = "Trial", 
       y     = "Accuracy", 
       title = "Human categorization") +
  scale_color_manual(values = c('#1b9e77')) + 
  plot_specs +
  theme(legend.position = "none")
```

## Figure 3c. Model unique feature performance

The network’s unique feature recognition performance when presented
with distinct categories of items with unique and shared features.

```
ggplot(m_ufm, aes(x = trial, y = accuracy, group = condition, color = condition))+
  geom_hline(yintercept = 0.25, 
             color      = "grey", 
             linetype   = "dashed", 
             size       = 1) +  
  geom_line(size = 1.5) +
  geom_errorbar(aes(ymin = accuracy - standard_error, 
                    ymax = accuracy + standard_error), 
                width    = .2,
                position = position_dodge(0.05), 
                size     = 1.2) +
  scale_x_continuous(limits = c(0, 140), 
                     breaks = c(0, 20, 40, 60, 80, 100, 120, 140)) +
  scale_y_continuous(limits = c(0.2, 1), 
                     breaks = c(0, .25, .5, .75, 1)) +
  scale_color_manual(values = c('#1b9e77','#d95f02','#7570b3')) +
  labs(x     = "Trial", 
       y     = "Accuracy",
       title = "Model unique feature performance") +
  plot_specs
```

## Figure 3d. Model categorization

The network’s categorization performance when presented with distinct
categories of items with unique and shared features.

```
ggplot(m_cat, aes(x = trial, y = accuracy, group = condition, color = condition))+
    geom_hline(yintercept = 0.33, 
               color = "grey", 
               linetype = "dashed", 
               size = 1) +
    geom_line(size = 1.5) +
  geom_errorbar(aes(ymin = accuracy - standard_error, 
                    ymax = accuracy + standard_error), 
                width    = .2,
                position = position_dodge(0.05), 
                size     = 1.2) +
  scale_x_continuous(limits = c(0, 140), 
                     breaks = c(0, 20, 40, 60, 80, 100, 120, 140)) +
  scale_y_continuous(limits = c(0.2, 1), 
                     breaks = c(0, .25, .5, .75, 1)) +
  scale_color_manual(values = c('#1b9e77','#d95f02','#7570b3')) +
  labs(x     = "Trial", 
       y     = "Accuracy",
       title = "Model categorization") +
  plot_specs
```

## Figure 3e. Model generalization

The network’s generalization performance when presented with distinct
categories of items with unique and shared features.

```
ggplot(m_gen, aes(x = trial, y = accuracy, group = condition, color = condition))+
  geom_hline(yintercept = 0.33, 
             color = "grey", 
             linetype = "dashed", 
             size = 1) +
   geom_line(size = 1.5) +
  geom_errorbar(aes(ymin = accuracy - standard_error, 
                    ymax = accuracy + standard_error), 
                width    = .2,
                position = position_dodge(0.05), 
                size     = 1.2) + 
  scale_x_continuous(limits = c(0, 140), 
                     breaks = c(0, 20, 40, 60, 80, 100, 120, 140)) +
  scale_y_continuous(limits = c(0.2, 1), 
                     breaks = c(0, .25, .5, .75, 1)) +
  scale_color_manual(values = c('#1b9e77','#d95f02','#7570b3')) +
  labs(x     = "Trial", 
       y     = "Accuracy",
       title = "Model generalization") +
  plot_specs
```

## Figure 3f. Representational similarity analysis: the initial vs settled response

Each item appears in the rows and columns of the heatmaps. The
diagonals are always 1, as this reflects items correlated to themselves,
and the off-diagonals are symmetric. Black boxes delineate
categories.

```
# Clear the environment
remove(list = ls())

# Define a function that converts a data frame to a matrix
matrix.please <- function(x) {
  m <- as.matrix(x[,-1])
  rownames(m) <- x[,1]
  m }

# Specify colors for heatmaps
dd <- (c('#a50026','#d73027','#f46d43','#fdae61','#fee090','#e0f3f8','#abd9e9','#74add1','#4575b4','#313695'))
pp <- rev(dd)

# Load the data
DG_initial <- read_excel("Figure 3 - Source Data 2.xlsx", sheet = "DG_MeanCorMatrix_test_init")
DG_settled <- read_excel("Figure 3 - Source Data 2.xlsx", sheet = "DG_MeanCorMatrix_test_set")

CA3_initial <- read_excel("Figure 3 - Source Data 2.xlsx", sheet = "CA3_MeanCorMatrix_test_init")
CA3_settled <- read_excel("Figure 3 - Source Data 2.xlsx", sheet = "CA3_MeanCorMatrix_test_set")

CA1_initial <- read_excel("Figure 3 - Source Data 2.xlsx", sheet = "CA1_MeanCorMatrix_test_init")
CA1_settled <- read_excel("Figure 3 - Source Data 2.xlsx", sheet = "CA1_MeanCorMatrix_test_set")
```

```
# Create a list containing all files
all_files <- list(DG_initial = DG_initial,   DG_settled = DG_settled,
                  CA3_initial = CA3_initial, CA3_settled = CA3_settled,
                  CA1_initial = CA1_initial, CA1_settled = CA1_settled)

# Initiate an empty list that to store plots  
plots_list <- list()
```

```
# Loop through the data for the initial and settled phase of each subfield, and visualize correlation matrices
for (i in 1:length(all_files)) {
  # load one dataset
  dat_temp = all_files[[i]]
  
  # extract information about the subfield (DG, CA3 and CA1) and phase (initial and settled)
  current_subfield <- gsub("_.*$","", names(all_files)[i])
  phase <- substr((names(all_files)[i]), 5, 7)
  # dynamically create a name for each plot
  temp_name <- paste("heatmap_", current_subfield, "_", phase, sep = "") 

  # rename and recode column names and row names  
  dat_temp <- dat_temp %>%
    rename(a1 = c1s2, a2 = c1s3, a3 = c1s4, a4 = c1s5,
           b1 = c2s2, b2 = c2s3, b3 = c2s4, b4 = c2s5,
           c1 = c3s2, c2 = c3s3, c3 = c3s4, c4 = c3s5) %>%
    mutate(item = recode_factor(item, "c1s2" = "a1", "c1s3" = "a2", "c1s4" = "a3", "c1s5" = "a4",
                                      "c2s2" = "b1", "c2s3" = "b2", "c2s4" = "b3", "c2s5" = "b4",
                                      "c3s2" = "c1", "c3s3" = "c2", "c3s4" = "c3", "c3s5" = "c4"))
  # define as data frame 
  dat_temp <- as.data.frame(dat_temp)
  
  # convert to a matrix
  dat_temp_M <- matrix.please(dat_temp)

 # produce a plot
  p <- ggplot(melt(dat_temp_M), aes(Var1, ordered(Var2, levels = rev(sort(unique(Var2)))), fill = value)) +
    geom_tile() +
    coord_equal() +
    scale_fill_gradientn(colours = pp, 
                         values = scales::rescale(c(-0.3, 0, 0.7)),
                         breaks = c(-0.2, 0, 0.2, 0.4, 0.6, 0.8, 1),
                         labels = c(-0.2, 0, 0.2, 0.4, 0.6, 0.8, 1),
                         limits = c(-0.3,1)) +
    ggtitle(paste(current_subfield)) +
    annotate("rect", xmin = 0.5, xmax = 4.5,  ymin = 8.5, ymax = 12.5, alpha = 0, color = "black", size = 1.2) +
    annotate("rect", xmin = 4.5, xmax = 8.5,  ymin = 4.5, ymax = 8.5,  alpha = 0, color = "black", size = 1.2) +
    annotate("rect", xmin = 8.5, xmax = 12.5, ymin = 0.5, ymax = 4.5,  alpha = 0, color = "black", size = 1.2) +
    annotate("rect", xmin = 0.5, xmax = 12.5, ymin = 0.5, ymax = 12.5, alpha = 0, color = "black", size = 0.3) +
    theme_void() +
    theme(legend.title = element_blank(),
          legend.text = element_text(size = 35), 
          legend.position = c("none"),
          plot.title = element_text(size = 30, hjust = 0.5), 
          panel.border = element_blank(),
          panel.background = element_blank(),
          panel.grid.major = element_blank(), 
          panel.grid.minor = element_blank(), 
          axis.title = element_blank(),
          axis.text = element_text(size = 20), 
          axis.text.x = element_text(size = 20), 
          axis.ticks = element_blank()) 
 
    plots_list[[i]] <- p 
}
```

```
# combine heatmaps into one figure
combined_heatmaps <- annotate_figure(ggarrange(
                             annotate_figure(ggarrange(plots_list[[1]], plots_list[[3]], plots_list[[5]],
                                       ncol = 3, nrow = 1), 
                                       left = text_grob("Initial response", 
                                       size = 30, rot = 90, hjust = 0.5)), 
                             annotate_figure(ggarrange(plots_list[[2]], plots_list[[4]], plots_list[[6]],
                                       ncol = 3, nrow = 1),  
                                       left = text_grob("Settled response", 
                                       size = 30, rot = 90, hjust = 0.5)),
                             ncol = 1, nrow = 2, 
                             widths = c(1, 1)), 
                             top = text_grob("Representations after learning", size = 40)) 


combined_heatmaps
```

```
# Plot legend 
as_ggplot(get_legend(
          ggplot(melt(dat_temp_M), aes(Var1, ordered(Var2, levels = rev(sort(unique(Var2)))), fill = value)) +
          geom_tile() +
          scale_fill_gradientn(colours = pp, 
                               values = scales::rescale(c(-0.3, 0, 0.7)),
                               breaks = c(-0.2, 0, 0.2, 0.4, 0.6, 0.8, 1),
                               labels = c(-0.2, 0, 0.2, 0.4, 0.6, 0.8, 1),
                               limits = c(-0.3,1)) +
          theme(legend.title = element_blank(),
                legend.text=element_text(size=16), 
                legend.position = c("left"))
))
```
