# Supplementary material for: A neural network model of hippocampal contributions to category learning
Source: eLife. 2023 Dec 11;12:e77185. doi: 10.7554/eLife.77185 (PMC10712951; doi:10.7554/eLife.77185)
Supplement: Figure 4—source code 1. [file elife-77185-fig4-code1.zip › 18-01-2022-RA-eLife-77185/Figure4Code.html]

A neural network model of hippocampal contributions to category learning


# A neural network model of hippocampal contributions to category learning

## Figure 4 - Source Code

This is an R code used to create Figure 4 for the paper “A neural
network model of hippocampal contributions to category learning”.

https://www.biorxiv.org/content/10.1101/2022.01.12.476051v1

```
# Load libraries
library(readxl)
library(dplyr)
library(ggplot2)
library(reshape2)
library(RColorBrewer)
library(ggpubr)
library(grid)
```

```
# Load data
data_fig4 <- as.data.frame(read.csv("Figure 4 - Source Data 1.csv"))

# Subset accuracy data for recognition test
rec <- data_fig4 %>%
  filter(test_type == "rec")

# Subset accuracy data for categorization test
cat <- data_fig4 %>%
  filter(test_type == "cat")
```

```
# define plot theme specs 
plot_specs <- 
  theme_bw() +
  theme(panel.grid.major = element_blank(), 
        panel.grid.minor = element_blank(),
        panel.border     = element_blank(),
        axis.line        = element_line(colour = "black"),
        axis.text        = element_text(size = 15),
        axis.title       = element_text(size = 20),
        plot.title       = element_text(size = 25), 
        legend.text      = element_text(size = 20),
        legend.title     = element_blank())
```

## Figure 4b. Model intact vs. lesioned categorization

Intact and lesioned model categorization performance across trials,
simulating the initial phase of human learning.

```
# average over MSP-only and TSP-only networks
les_avg <- cat %>%
  mutate(Condition_Amn = ifelse(condition == 'Intact', 
                                'Intact', 'Average MSP-TSP lesion')) %>%
  group_by(Condition_Amn, trial) %>%
  summarise(accuracy_mean = mean(accuracy),
            se = mean(standard_error))  

# re-order factor levels 
les_avg$Condition_Amn <- factor(les_avg$Condition_Amn, levels=c('Intact', 'Average MSP-TSP lesion'))

# plot
ggplot(les_avg, aes(x = trial, y = accuracy_mean, linetype = Condition_Amn)) +
  geom_line(size = 2) +
  geom_errorbar(aes(ymin = accuracy_mean-se, 
                    ymax = accuracy_mean+se), 
                width    = .2,
                position = position_dodge(0.05), 
                size     = 1.2) + 
  scale_x_continuous(breaks = seq(0, 50, 10)) +
  scale_y_continuous(limits = c(0.45, 0.75), 
                     breaks = seq(0.45, 0.75, 0.05),
                     labels = c(45, 50, 55, 60, 65, 70, 75)) + 
  geom_hline(yintercept=0.50, 
             linetype="dashed", 
             color = "grey") +
  labs(x = "Trial", 
       y = "Percent Correct",
       title = "Model intact vs. lesioned categorization") +
  plot_specs + 
  theme(legend.position = "right")
```

```
## Warning: Using `size` aesthetic for lines was deprecated in ggplot2 3.4.0.
## ℹ Please use `linewidth` instead.
## This warning is displayed once every 8 hours.
## Call `lifecycle::last_lifecycle_warnings()` to see where this warning was
## generated.
```

## Figure 4c. Recognition accuracy

The network’s recognition performance when presented with
probabilistic categories.

```
  ggplot(rec, aes(x = trial, y = accuracy, colour = condition)) +
  geom_hline(yintercept = 0.50, 
             linetype   = "dashed", 
             color      = "grey") +
  geom_line(size = 1.5) +
  geom_errorbar(aes(ymin = accuracy - standard_error, 
                    ymax = accuracy + standard_error), 
                width    = .2,
                position = position_dodge(0.05), 
                size     = 1.2) + 
  scale_x_continuous(breaks = seq(0, 50, 10)) +
  scale_y_continuous(limits = c(0.4, 0.8), 
                     breaks = seq(0.4, 0.8, 0.10)) + 
  scale_color_manual(values = c('#1b9e77','#d95f02','#7570b3')) +
  labs(x     = "Trial", 
       y     = "Accuracy", 
       title = "Model recognition") +
  plot_specs
```

## Figure 4d. Categorization performance

The network’s categorization performance when presented with
probabilistic categories.

```
 ggplot(cat, aes(x = trial, y = accuracy, colour = condition)) +
  geom_hline(yintercept = 0.50, 
             linetype   = "dashed", 
             color      = "grey") +
  geom_line(size = 1.5) +
  geom_errorbar(aes(ymin = accuracy - standard_error, 
                    ymax = accuracy + standard_error), 
                width    = .2,
                position = position_dodge(0.05), 
                size     = 1.2) + 
  scale_x_continuous(breaks = seq(0, 50, 10)) +
  scale_y_continuous(limits = c(0.4, 0.8), 
                     breaks = seq(0.4, 0.8, 0.10)) + 
  scale_color_manual(values = c('#1b9e77','#d95f02','#7570b3')) +
  labs(x     = "Trial", 
       y     = "Accuracy", 
       title = "Model categorization") +
  plot_specs
```

## Figure 4e. Model representations after learning: representational similarity analysis

```
# Clear the environment
remove(list = ls())

# Define a function that converts a data frame to a matrix
matrix.please <- function(x) {
  m <- as.matrix(x[,-1])
  rownames(m) <- x[,1]
  m }

# Specify colors for heatmaps
dd <- (c('#a50026','#d73027','#f46d43','#fdae61','#fee090','#e0f3f8','#abd9e9','#74add1','#4575b4','#313695'))
pp <- rev(dd)

# Load the data
DG_initial <- read_excel("Figure 4 - Source Data 2.xlsx", sheet = "DG_MeanCorMatrix_test_init")
DG_settled <- read_excel("Figure 4 - Source Data 2.xlsx", sheet = "DG_MeanCorMatrix_test_set")

CA3_initial <- read_excel("Figure 4 - Source Data 2.xlsx", sheet = "CA3_MeanCorMatrix_test_init")
CA3_settled <- read_excel("Figure 4 - Source Data 2.xlsx", sheet = "CA3_MeanCorMatrix_test_set")

CA1_initial <- read_excel("Figure 4 - Source Data 2.xlsx", sheet = "CA1_MeanCorMatrix_test_init")
CA1_settled <- read_excel("Figure 4 - Source Data 2.xlsx", sheet = "CA1_MeanCorMatrix_test_set")
```

```
# Create a list containing all files
all_files <- list(DG_initial = DG_initial,   DG_settled = DG_settled,
                  CA3_initial = CA3_initial, CA3_settled = CA3_settled,
                  CA1_initial = CA1_initial, CA1_settled = CA1_settled)

# Initiate an empty list that will store plots  
plots_list <- list()
```

```
# Loop through the data for the initial and settled phase of each subfield, and visualize correlation matrices
for (i in 1:length(all_files)) {
  # load one dataset
  dat_temp = all_files[[i]]
  
  # extract information about the subfield (DG, CA3 and CA1) and phase (initial and settled)
  current_subfield <- gsub("_.*$","", names(all_files)[i])
  phase <- substr((names(all_files)[i]), 5, 7)
  # dynamically create a name for each plot
  temp_name <- paste("heatmap_", current_subfield, "_", phase, sep = "") 

  # define as data frame 
  dat_temp <- as.data.frame(dat_temp)
  
  # convert to a matrix
  dat_temp_M <- matrix.please(dat_temp)

 # produce a plot
  p <- ggplot(melt(dat_temp_M), aes(Var1, ordered(Var2, levels = rev(sort(unique(Var2)))), fill = value)) +
          geom_tile() +
          coord_equal() +
          scale_fill_gradientn(colours = pp, 
                               values = scales::rescale(c(-0.2, 1)),
                               breaks=c(-0.2, 0, 0.2, 0.4, 0.6, 0.8, 1),
                               labels=c(-0.2, 0, 0.2, 0.4, 0.6, 0.8, 1),
                               limits=c(-0.2, 1)) + 
    ggtitle(paste(current_subfield)) +
          annotate("rect", xmin = 0.5, xmax = 6.5, ymin = 6.5, ymax = 12.5, alpha = 0, color = "black", size = 1.2) +
          annotate("rect", xmin = 6.5, xmax = 12.5, ymin = 0.5, ymax = 6.5, alpha = 0, color = "black", size = 1.2) +
          theme_void() +
          theme(legend.title = element_blank(),
          legend.text = element_text(size = 35), 
          legend.position = c("none"),
          plot.title = element_text(size = 30, hjust = 0.5), 
          panel.border = element_blank(),
          panel.background = element_blank(),
          panel.grid.major = element_blank(), 
          panel.grid.minor = element_blank(), 
          axis.title = element_blank(),
          axis.text = element_blank(), 
          axis.text.x = element_blank(),
          axis.ticks = element_blank()) 
  
  
    plots_list[[i]] <- p 
}
```

```
# combine the heatmaps into one figure
combined_heatmaps <- annotate_figure(ggarrange(
                             annotate_figure(ggarrange(plots_list[[1]], plots_list[[3]], plots_list[[5]],
                                       ncol = 3, nrow = 1), 
                                       left = text_grob("Initial response", 
                                       size = 40, rot = 90, hjust = 0.5)), 
                             annotate_figure(ggarrange(plots_list[[2]], plots_list[[4]], plots_list[[6]],
                                       ncol = 3, nrow = 1),  
                                       left = text_grob("Settled response", 
                                       size = 40, rot = 90, hjust = 0.5)),
                             ncol = 1, nrow = 2, 
                             widths = c(1, 1)), 
                             top = text_grob("Model representations after learning", size = 50)) 


combined_heatmaps
```

```
# Plot legend 
as_ggplot(get_legend(
          ggplot(melt(dat_temp_M), aes(Var1, ordered(Var2, levels = rev(sort(unique(Var2)))), fill = value)) +
          geom_tile() +
 scale_fill_gradientn(colours = pp, 
                               values = scales::rescale(c(-0.2, 1)),
                               breaks=c(-0.2, 0, 0.2, 0.4, 0.6, 0.8, 1),
                               labels=c(-0.2, 0, 0.2, 0.4, 0.6, 0.8, 1),
                               limits=c(-0.2, 1)) + 
          theme(legend.title = element_blank(),
                legend.text=element_text(size=16), 
                legend.position = c("left"))
))
```
