# Supplementary material for: A neural network model of hippocampal contributions to category learning
Source: eLife. 2023 Dec 11;12:e77185. doi: 10.7554/eLife.77185 (PMC10712951; doi:10.7554/eLife.77185)
Supplement: Figure 5—source code 1. [file elife-77185-fig5-code1.zip › 18-01-2022-RA-eLife-77185/Figure5Code.html]

A neural network model of hippocampal contributions to category learning


# A neural network model of hippocampal contributions to category learning

## Figure 5 - Source Code

This is an R code used to create Figure 5 for the paper “A neural
network model of hippocampal contributions to category learning”.

https://www.biorxiv.org/content/10.1101/2022.01.12.476051v1

```
# Load libraries
library(readxl)
library(dplyr)
library(ggplot2)
library(reshape2)
library(RColorBrewer)
library(ggpubr)
library(grid)
library(forcats)
```

```
# Load data
data_fig5cd <- as.data.frame(read.csv("Figure 5 - Source Data 1.csv"))

data_fig5ef <- as.data.frame(read.csv("Figure 5 - Source Data 2.csv"))

# Define order of levels for the factor "atypical_features"
data_fig5ef <- data_fig5ef %>%
  mutate(atypical_features = fct_relevel(atypical_features, 
                                         "4 atypical", "3 atypical", "2 atypical", "1 atypical"))
  
# Subset accuracy data for recognition test
rec <- data_fig5ef %>%
  filter(test_type == "rec")

# Subset accuracy data for generalization test
gen <- data_fig5ef %>%
  filter(test_type == "gen")
```

```
# define plot theme specs 
plot_specs <- 
  theme_bw() +
  theme(panel.grid.major = element_blank(), 
        panel.grid.minor = element_blank(),
        panel.border     = element_blank(),
        axis.line        = element_line(colour = "black"),
        axis.text        = element_text(size = 15),
        axis.title       = element_text(size = 20),
        plot.title       = element_text(size = 25), 
        legend.text      = element_text(size = 20),
        legend.title     = element_blank())
```

## Figure 5c. Model generalization across learning

Intact model generalization across learning, with 10 trials prior to
each interim test.

```
m_gen_1 <- data_fig5cd %>% 
  filter(test_counter %in% c(5, 15, 25, 35))

# re-order factor levels 
m_gen_1$AtypFeat_r <- factor(m_gen_1$AtypFeat_r, levels=c( "Prototypes", 
                                                           "Distance 1", 
                                                           "Distance 2", 
                                                           "Distance 3", 
                                                           "Distance 4"))

# plot
ggplot(m_gen_1, aes(x = test_counter, y = Accuracy, group = AtypFeat_r, color = AtypFeat_r)) +
  geom_line(size = 3) +
  geom_errorbar(aes(ymin = Accuracy - se, 
                    ymax = Accuracy + se), 
                width    = .2,
                position = position_dodge(0.05)) +
  scale_x_continuous(breaks   = c(5, 15, 25, 35),
                     n.breaks = 4,
                     limits   = c(0, 40),
                     labels   = c(1, 2, 3, 4)) + 
  scale_color_manual(values = c('#252525', '#525252','#737373','#969696','#bdbdbd')) +
  labs(x     = "Interim test number", 
       y     = "Proportion correct",
       title = "Model generalization across learning") + 
  coord_cartesian(ylim = c(0.5, 1)) + 
  plot_specs
```

## Figure 5d. Model generalization

Intact model generalization at the end of learning.

```
# select final test
m_gen_2 <- data_fig5cd %>% 
  filter(test_counter == 40)

# plot
ggplot(m_gen_2, aes(x = AtypFeat, y = Accuracy)) +
  geom_bar(stat = "identity", width = 0.5, color = "black", fill = "#737373") +
  geom_errorbar(aes(ymin = Accuracy-se, 
                    ymax = Accuracy+se), 
                width    = .2,
                position = position_dodge(.9)) +
  labs(x     = "Distance from prototypes", 
       y     = "Proportion correct",
       title = "Model generalization") + 
  coord_cartesian(ylim = c(0.5, 1)) + 
  plot_specs
```

## Figure 5e. Generalization

Model generalization broken down by typicality and model type.

```
ggplot(gen, aes(x = trial, y = accuracy, group = condition, color = condition)) +
  geom_line(size = 2) +
  geom_errorbar(aes(ymin = accuracy - standard_error, 
                    ymax = accuracy + standard_error), 
                width    = .2,
                position = position_dodge(0.05)) +
  scale_x_continuous(breaks = seq(0, 100, 20)) + 
  scale_color_manual(values = c('#1b9e77','#d95f02','#7570b3')) +
  labs(x     = "Trial", 
       y     = "Accuracy",
       title = "Model generalization") + 
  geom_hline(yintercept = 0.5, linetype = "dashed", colour = "grey") +
  coord_cartesian(ylim = c(0.3, 1)) + 
  facet_grid(~ atypical_features) +
  theme_bw() +
  theme(panel.grid.major = element_blank(), 
        panel.grid.minor = element_blank(),
        panel.border     = element_blank(),
        axis.line        = element_line(colour = "black"),
        axis.text        = element_text(size=20),
        axis.title       = element_text(size=30),
        legend.position  = "none",
        legend.title     = element_blank(),
        strip.text.x     = element_text(size = 30),
        plot.title       = element_text(size = 40, hjust = 0.5),
        strip.background = element_blank())
```

## Figure 5f. Atypical feature recognition

Model atypical feature recognition broken down by typicality and
model type.

```
ggplot(rec, aes(x = trial, y= accuracy, group = condition, color = condition)) +
  geom_line(size = 2) +
  geom_errorbar(aes(ymin = accuracy - standard_error, 
                    ymax = accuracy + standard_error), 
                width    = .2,
                position = position_dodge(0.05)) + 
  scale_x_continuous(breaks = seq(0, 100, 20)) + 
  labs(x     = "Trial", 
       y     = "Accuracy", 
       title = "Model atypical feature recognition") + 
  geom_hline(yintercept = 0.5, linetype = "dashed", colour = "grey") +
  scale_color_manual(values = c('#1b9e77', '#d95f02', '#7570b3', '#bcbddc')) +
  coord_cartesian(ylim = c(0.3, 1)) + 
  facet_wrap(~ atypical_features, nrow = 1) +
  theme_bw() +
  theme(panel.grid.major = element_blank(), 
        panel.grid.minor = element_blank(),
        panel.border     = element_blank(),
        axis.line        = element_line(colour = "black"),
        axis.text        = element_text(size = 20),
        axis.title       = element_text(size = 30),
        plot.subtitle    = element_text(hjust = 0.5, size=30),
        plot.title       = element_text(size = 40, hjust = 0.5),
        legend.text      = element_text(size = 20),
        legend.position  = c("right"),
        legend.title     = element_blank(),
        legend.key.width = unit(3, "line"),
        strip.background = element_blank(),
        strip.text       = element_text(size = 30),
        panel.spacing    = unit(2, "lines"))
```

## Figure 5g. Model representations after learning: representational similarity analysis

Representational similarity for the initial and settled response of
the intact network. Each item appears in the rows and columns of the
heatmap, organized by most prototypical members of one category to most
prototypical members of the other.The diagonals are always 1, as this
reflects items correlated to themselves, and the off-diagonals are
symmetric. Black boxes delineate categories.

```
# Clear the environment
remove(list = ls())

# Define a function that converts a data frame to a matrix
matrix.please <- function(x) {
  m <- as.matrix(x[,-1])
  rownames(m) <- x[,1]
  m }

# Specify colors for heatmaps
dd <- (c('#a50026','#d73027','#f46d43','#fdae61','#fee090','#e0f3f8','#abd9e9','#74add1','#4575b4','#313695'))
pp <- rev(dd)

# Load the data
DG_initial <- read_excel("Figure 5 - Source Data 3.xlsx", sheet = "DG_MeanCorMatrix_test_init")
DG_settled <- read_excel("Figure 5 - Source Data 3.xlsx", sheet = "DG_MeanCorMatrix_test_set")

CA3_initial <- read_excel("Figure 5 - Source Data 3.xlsx", sheet = "CA3_MeanCorMatrix_test_init")
CA3_settled <- read_excel("Figure 5 - Source Data 3.xlsx", sheet = "CA3_MeanCorMatrix_test_set")

CA1_initial <- read_excel("Figure 5 - Source Data 3.xlsx", sheet = "CA1_MeanCorMatrix_test_init")
CA1_settled <- read_excel("Figure 5 - Source Data 3.xlsx", sheet = "CA1_MeanCorMatrix_test_set")
```

```
# Create a list containing all files
all_files <- list(DG_initial  = DG_initial,   DG_settled = DG_settled,
                  CA3_initial = CA3_initial, CA3_settled = CA3_settled,
                  CA1_initial = CA1_initial, CA1_settled = CA1_settled)

# Initiate an empty list that will store plots  
plots_list <- list()
```

```
# Loop through the data for the initial and settled phase of each subfield, and visualize correlation matrices
for (i in 1:length(all_files)) {
  # load one dataset
  dat_temp = all_files[[i]]
  
  # extract information about the subfield (DG, CA3 and CA1) and phase (initial and settled)
  current_subfield <- gsub("_.*$","", names(all_files)[i])
  phase <- substr((names(all_files)[i]), 5, 7)
  # dynamically create a name for each plot
  temp_name <- paste("heatmap_", current_subfield, "_", phase, sep = "") 

  # define as data frame 
  dat_temp <- as.data.frame(dat_temp)
  
  # convert to a matrix
  dat_temp_M <- matrix.please(dat_temp)

 # produce a plot
  p <- ggplot(melt(dat_temp_M), aes(Var1, ordered(Var2, levels = rev(sort(unique(Var2)))), fill = value)) +
    geom_tile() +
    coord_equal() +
    scale_fill_gradientn(colours = pp, 
                         values  = scales::rescale(c(-0.04, 1)), 
                         breaks  = c(0, 0.2, 0.4, 0.6, 0.8, 1),
                         labels  = c(0, 0.2, 0.4, 0.6, 0.8, 1),
                         limits  = c(-0.04,1)) +
    ggtitle(paste(current_subfield)) +
    annotate("rect", xmin = 0.5, xmax = 42.5, ymin = 0.5, ymax = 42.5, alpha = 0, color = "black", size = 0.3) +
    annotate("rect", xmin = 0.5, xmax = 21.5, ymin = 21.5, ymax = 42.5, alpha = 0, color = "black", size = 1.2) +
    annotate("rect", xmin = 21.5, xmax = 42.5,  ymin = 0.5, ymax = 21.5, alpha = 0, color = "black", size = 1.2) +
    theme_void() +
    theme(legend.title     = element_blank(),
          legend.text      = element_text(size = 35), 
          legend.position  = c("none"),
          plot.title       = element_text(size = 30, hjust = 0.5), 
          panel.border     = element_blank(),
          panel.background = element_blank(),
          panel.grid.major = element_blank(), 
          panel.grid.minor = element_blank(), 
          axis.title       = element_blank(),
          axis.text        = element_blank(), 
          axis.ticks       = element_blank()) 
 
    plots_list[[i]] <- p 
}
```

```
# combine the heatmaps into one figure
combined_heatmaps <- annotate_figure(ggarrange(
                             annotate_figure(ggarrange(plots_list[[1]], plots_list[[3]], plots_list[[5]],
                                       ncol = 3, nrow = 1), 
                                       left = text_grob("Initial response", 
                                       size = 30, rot = 90, hjust = 0.5)), 
                             annotate_figure(ggarrange(plots_list[[2]], plots_list[[4]], plots_list[[6]],
                                       ncol = 3, nrow = 1),  
                                       left = text_grob("Settled response", 
                                       size = 30, rot = 90, hjust = 0.5)),
                             ncol = 1, nrow = 2, 
                             widths = c(1, 1)), 
                             top = text_grob("Model representations after learning", size = 40)) 


combined_heatmaps
```

```
# Plot legend 
as_ggplot(get_legend(
          ggplot(melt(dat_temp_M), aes(Var1, ordered(Var2, levels = rev(sort(unique(Var2)))), fill = value)) +
          geom_tile() +
          scale_fill_gradientn(colours = pp, 
                         values = scales::rescale(c(-0.04, 1)), 
                         breaks = c(0, 0.2, 0.4, 0.6, 0.8, 1),
                         labels = c(0, 0.2, 0.4, 0.6, 0.8, 1),
                         limits = c(-0.04,1)) +
          theme(legend.title = element_blank(),
                legend.text = element_text(size = 16), 
                legend.position = c("left"))
))
```
